# Bringing It All Together: A Review of the Challenges in Measuring Children’s Satisfaction as a Key Component of Acute Pain Management

**DOI:** 10.3390/children7110243

**Published:** 2020-11-20

**Authors:** Joseph W. Hodapp, Samina Ali, Amy L. Drendel

**Affiliations:** 1 Department of Anesthesiology, Stanford University, Pal Alto, CA 94305, USA; hodappjw@gmail.com; 2 Department of Pediatrics, Faculty of Medicine & Dentistry, University of Alberta, Edmonton, AB T6G 2R3, Canada; sali@ualberta.ca; 3 Department of Pediatrics, Section of Emergency Medicine, Medical College of Wisconsin, Milwaukee, WI 53201, USA

**Keywords:** pediatric, satisfaction, pain, pain management

## Abstract

In 2008, the Pediatric Initiative on Methods, Measurement, and Pain Assessment in Clinical Trials (PedIMMPACT) published a consensus statement that recognized the dearth of research surrounding the topic of children’s satisfaction with acute pain management. This review of published literature will summarize what is known about the topic of children’s satisfaction with pain management, identify current gaps in the knowledge, and provide direction for future research in this critical area. Including children in the decision-making process as soon as they are developmentally able is a concept that is the fundamental basis for seeking assent and more active roles within healthcare decisions for children. It is the responsibility of adults to provide them with increasing opportunities for self-evaluation and more independent management of their healthcare, encouraging the development of children into adults. As clinicians and researchers, it is our prerogative to support the maturation of children by building effective methods to communicate their satisfaction with acute pain treatment and healthcare. Children’s satisfaction with acute pain management is not well studied and further research is needed for the development of inclusive, developmentally appropriate measures of satisfaction for our pediatric patients.

## 1. Introduction

In 2008, the Pediatric Initiative on Methods, Measurement, and Pain Assessment in Clinical Trials (PedIMMPACT) published a consensus statement that included eight core outcomes in clinical trials of pain treatment of children. Among these was global satisfaction with pain management, an understudied area for which they were unable to provide an evidence-based recommendation [1]. Indeed, less than 20% of published interventional trials for postoperative pediatric pain include satisfaction as an outcome measure [2]. A growing body of medical research demonstrates that school-age children are capable of validly and reliably assessing their own healthcare experience [1,2,3,4,5,6,7,8,9,10], that using proxy assessments of a child’s healthcare may not provide a complete picture of the child’s own experience [11,12], that children and parents often disagree on what would improve their pain management [13,14], and that children as young as 18 months have the language to speak about their own subjective experience of acute pain [15]. Despite this knowledge, the voice of the child patient remains limited in the evaluation of their own treatment [16]. As such, there is no “best practice” measure that offers a standardized method to assess pediatric perceptions of their own acute pain management.

The objective of this paper is to highlight what is known about the topic of satisfaction, specifically as it pertains to pain management, identify the specific gaps in our knowledge of pediatric satisfaction in regard to pain management, and provide a foundation on which to direct future research in this critical area. In the last several years, multiple comprehensive literature reviews of both satisfaction [17,18,19,20,21,22] and acute pediatric pain assessment [23,24] have been published; our goal is not to provide yet another comprehensive review, but rather a strategic synthesis of what is known in order to provide a clear direction for future research and clinical quality assurance programs. This focus article will provide a call to action, such that we are no longer talking about why pediatric input with acute pain management is important, but rather determining how we may begin to close the gap that exists between what is considered best practice and what is happening in the clinical setting. Health professionals should recognize that young patients ought to be given a voice in their care which will provide a richer understanding of the overall acute pain experience for children. It is the goal of this focus article to be the first of many analyses and clinical efforts to formulate and implement a globally effective measure of pediatric satisfaction with acute pain management.

## 2. What Do We Know about Satisfaction with Acute Pain Management?

### 2.1. Why Is Satisfaction an Important Measure?

In the last thirty years within the healthcare industry, satisfaction has grown as a vital measure by which to evaluate healthcare, caregivers, and the patient population itself. In an age where paternalistic medicine is declining, and the patient’s voice has grown in importance, satisfaction has become an important measure in the assessment of healthcare and is intimately tied to, but certainly not synonymous with, quality of care [25,26]. It has been shown to be connected to patients’ expectations for care [27,28] and has a profound impact on the professional reputation and financial status of an institution [25,29]. Increased willingness to return [30], medical compliance [31], and a decrease in malpractice litigation are just a few measurable outcomes that have been positively linked to satisfaction [29,30]. As the patient’s voice has gained strength over the last several decades, directly measuring satisfaction provides an opportunity for our healthcare system to circumvent provider bias and paint a more complete picture of treatment perceptions of the patients themselves [32]. Further, it allows us to tap into patients’ perceptions and values regarding their medical care [33] and bridges the gap between the healthcare system and the population it seeks to treat by evaluating the process of care [18]. As viewing medical care “through the patient’s eyes” remains an ethical and professional imperative [34], measuring patient satisfaction as a fundamental element of the dialogue between the patient population and the healthcare system becomes essential. When children are the patients, there is an opportunity for self-evaluation and, depending on the age of the child, some independent management of their own healthcare, so measuring the satisfaction of the pediatric patient is important as we encourage a child’s development.

### 2.2. What Do We Know about Satisfaction?

Having established the importance of satisfaction as an evaluative measure of healthcare, our first challenge is how to go about collecting satisfaction data from patients in a reliable and timely manner. This challenge is two-fold, as we must both accurately define satisfaction and determine how to accurately measure it in the clinical setting. The uptake of measuring satisfaction was so rapidly embraced in the adult clinical world—where in some settings it was considered the most desirable outcome in medical care—that little attention was paid to its theoretical construct [26]. Taking a step back, it is vital to start with a proper working definition. Satisfaction is both a cognitive and affective response to salient aspects of care [17,19] that is influenced by a patient’s preferences and expectations [32,35]. It is highly subjective and multidimensional, being both a measure of the healthcare provider, healthcare service provided, and of the patient’s values and goals with respect to their care [35]. The aspects of care that drive patient satisfaction are the components of a global assessment of satisfaction that are important to consider when defining satisfaction.

In the last several decades, there have been countless studies attempting to clarify which aspects of care are most closely associated with high levels of satisfaction. Ware [35] laid a foundation for current satisfaction research, delineating eight aspects of care deemed to be most intimately connected to satisfaction: interpersonal manner of the providers, technical quality of care, accessibility/convenience, finances, efficacy/outcomes of care, continuity of care, physical environment, and availability of medical resources [35]. Much of the modern satisfaction research has come from these eight pillars, although there is still much debate as to the importance of each characteristic and the weight of its individual effect on measured satisfaction. It is not known whether the pediatric patient will prioritize characteristics differently than their parents. A review of the literature demonstrates that positive interactions and communications with members of the healthcare team are consistently the most highly correlated with adult patient satisfaction [21,36,37,38,39,40,41,42]. Results from the 2016 Press Ganey EDCAHPS Early Adopter Study further defend the assertion that provider support forms a crucial component of the pain management experience, demonstrating that empathic listening was more significantly correlated with satisfaction than receiving medication [43]. As the field of pediatric satisfaction with pain management expands and our knowledge grows, it will be crucial to keep these dimensions in mind for the development and validation of measurement tools for clinicians and researchers. These are the components of a global assessment of satisfaction for children that can aid in measuring validity.

### 2.3. What Do We Know about Pediatric Satisfaction with Pain Management?

In 1995, the American Pain Society labeled satisfaction with pain management as one of the most important types of outcome data [44]. Two decades of satisfaction research have affirmed that overall satisfaction with healthcare is highly correlated with pain relief in adult clinical trials [21,45,46,47] and in the pediatric emergency department [38,41,48,49,50,51]. Despite the common use of parents/guardians as proxy reports in the measurement of pediatric pain or the use of observational methods, these methods tend to underestimate both pain score [3,52,53,54,55] and pain resolution after treatment [38] compared to a child’s self-report. Additionally, a comparison of child and parent satisfaction in an ambulatory pediatric clinic demonstrated significantly different satisfaction ratings between the two parties, highlighting the importance of a pediatric satisfaction measure. Prior studies have shown parents express higher satisfaction than their children and marked differences in the qualitative assessments of the care experience [12]. In a study of parent–child pairs in a pediatric emergency department, Magaret et al. [38] demonstrated that satisfaction is positively correlated with the quality of provider–patient interactions, the sharing of adequate information, and the resolution of pain—all measures which require a great deal of subjective reporting. Armed with this knowledge, the PedIMMPACT committee reinforced the imperative to design and execute a standardized measure of pediatric satisfaction with pain management that is based on child self-reporting, removing the confounding factor of potentially incomplete parent proxy reports and creating space for children to tap into their own subjective evaluation of their healthcare experience [1].

Since the PedIMMPACT consensus in 2008, important strides have been made in the field of pediatric satisfaction. Weingarten et al. [50] examined pediatric satisfaction of pain management in an emergency department utilizing a modified version of the Total Quality Pain Management Questionnaire by Foster and Varni [50,56]. Using a population of 7 to 17-year-old patients, they found for children that pain resolution, provider interactions, and the perception that the medicine worked quickly had the strongest influence on satisfaction [50]. Compared to their parents’ assessment, pediatric patients reported having pain more frequently, that the medicine took a longer time to work, that the medication did not taste as bad as their parents thought it did, that they would request the same medicine more often, and that their most severe pain was rated more highly [13]. These recent studies further confirm the need for the development and introduction of a standardized satisfaction measure based on self-reporting that is congruent with a child’s developmental level and able to account for the culture and context of the pain experience in order to have a richer understanding of satisfaction with acute pain treatment [57].

Another consideration when creating and implementing a pediatric satisfaction scale related to the pain experience is the fact that children and adults alike consistently provide high ratings of satisfaction with pain management, despite continuing to experience high levels of pain [44,50,51,58,59]. This is in contrast to studies that have shown that satisfaction and pain reduction are highly correlated [21,38,41,45,46,47,48,49,50,51]. It has been postulated that this inconsistency in the literature is because some patients may expect to experience the common “peak and trough” nature of pain management—when pain levels increase, analgesics are administered to decrease the pain, resulting in times where the pain is not well controlled. Thus, the expectation is not for true sustained pain relief, but rather for periodic/cyclic pain decrease over time and with proper analgesia [58]. As we move towards a more holistic measure of a pediatric patient’s acute pain management experience, satisfaction measures will likely need to be enhanced by in-depth questions on many aspects of pain treatment including, but not limited to, technical quality of care measured by pain severity and emotional/functional impact, but pediatric patients may have other priorities that influence their satisfaction that must be addressed to avoid providing suboptimal care through inadequately treated pain [44].

## 3. Challenges in Developing a Measure of Pediatric Satisfaction with Pain Management

As a society and a healthcare system, we strive to include children in the decision-making process as soon as they are developmentally able, a concept that is the fundamental basis behind seeking assent and more active roles within healthcare decisions for children. As children develop and learn, they are provided with increasing opportunities for self-evaluation and more independent management of their healthcare, thereby encouraging their development into adults [60]. As clinicians and researchers, it is our prerogative to support the maturation of children by building effective methods to communicate their satisfaction with pain treatment and healthcare in general [61,62]. Currently, there are no validated tools to assess satisfaction from younger children.

### 3.1. General Challenges with Assessing Satisfaction with Pain Management

It has been demonstrated that most children aged four and older are developmentally able to use standard self-reports of pain, demonstrating an understanding of pain as a subjective experience [1,57]. Of note, however, most children do not consistently understand the meaning of satisfaction until 13 years of age [14,63]. For children over 13 years of age, the typical measures used by adults to measure satisfaction may be appropriate. The challenge for clinicians and researchers alike lies in best determining how to appropriately ask a pre-adolescent or younger child not only what their pain experience (e.g., severity, impact) was like, but how well their pain was managed (e.g., satisfaction, quality assessment).

We propose that a socio-communication model of pain be used in order to create the best tool to measure child satisfaction with pain management. Cognitive and social processes play an important role in experiencing and responding to pain and this model integrates an understanding of the social determinants of pain with psychological and biological systems that are important to consider when measuring satisfaction. This model is well suited to tool development because it appreciates pain as a form of social behavior, heavily influenced by external and internal factors [64,65]. The multidimensional nature of satisfaction is also well matched to this model [18,35,65,66]. The concept of satisfaction is impossible to distill to a single dimension, including evaluations regarding effectiveness, efficiency, and equality of the structure, process, or outcome of treatment [18]. This makes tool creation particularly difficult considering that children have age-dependent developmental differences to consider. In fact, likely multiple measures may be needed given the developmental differences between children of different ages and cognitive abilities. Additionally, the generally high rates of satisfaction noted in the literature regarding pain management [44] and the tendency for pediatric patients to respond extremely when ranking emotional states [67,68,69] raise the challenge of having to contend with falsely elevated satisfaction ratings. This has proven to be problematic across the spectrum of satisfaction research [38,47,70,71], leading Larsen et al. [32] to suggest that dissatisfaction data are far more important and reflective on the healthcare process than satisfaction data.

Clinicians, for example, nurses, psychologists, social workers, and physicians, are also faced with the problem of choosing how to administer a survey to measure satisfaction with pediatric pain management. The most common types of administration include: in person, by telephone, or mail-out with return response, each with its own limitations, especially when the age of the respondents is considered [19]. For adult patients, internal administration has returned both higher scores and higher response rates, compared to external/mailed surveys—with the exception of online surveys [72,73]. An internal or mailed survey would likely provide the highest yield, considering the inability to use successful visual indices of pain via telephone interviews [57,74,75,76]. However, when dealing with children of all ages, access to a telephone and the level of responsibility/accountability for a child will vary greatly across age groups and limit direct contact outside of the healthcare visit. This challenge can be best addressed by utilizing a point-of-care survey, which would eliminate these barriers. Nurses have an important role in the measurement and treatment of pain, and as such, a satisfaction measure will likely capture this.

### 3.2. Finding the Appropriate Items to Measure

In an effort to construct an accessible, developmentally conscious measure of pediatric satisfaction with pain management, the PedIMMPACT committee suggested the use of a single, global assessment [1]. Global ratings of satisfaction have been used in the adult literature regarding analgesia, with similar efficacy compared to more complex measures [46,77]. However, the downfall of the currently used global assessments for satisfaction is that they require parental response and do not directly engage the child in the assessment of their care. Nevertheless, the dimensions considered in the suggested global assessments are noteworthy: pain relief, symptoms, side effects, physical recovery, emotional recovery, participation in their roles as a student and family member, sleep, and economic considerations [1]. Amidst the current opioid crisis, we have learned from acute pain management in adults that functional improvements should be prioritized as a core dimension of the global assessment. Now, derived from the framework suggested by this expert committee, we can utilize these dimensions as a launching point to create a satisfaction measurement tool that will be relevant to the child patient. Whether a single global rating of satisfaction can be used for the pediatric patient is not known. Given the complexity of satisfaction, there may be a need for more than a single global rating suggested by the consensus statement.

We suggest that any future satisfaction measure be formatted to include visual markers, accompanied by developmentally appropriate language further discussed below for younger children. Faces scales present a possible solution to this because, in contrast to the visual analog scale (VAS) and numeric pain rating scale (NRS), they do not require children to be able to seriate or estimate magnitude. Thus, the development of a validated faces scale would allow for evaluation of satisfaction with young patients, as these skills are generally not present in pre-school children [69,78,79]. Though faces scale have been shown to be appropriate for older children and adolescents, it is not clear whether this would be the best approach for older children that have been shown to be able to report experiences using the scales intended for adults. Similar to the pain assessment tools, a different satisfaction assessment scale may need to be developed for older youth and adolescents to account for varying skills and cognitive abilities. It is possible that multiple measures will be needed to assess satisfaction for the pediatric patient. This also allows for the child to choose the measure they prefer to use.

### 3.3. Developing the Appropriate Scale

#### 3.3.1. Considerations for a Developmentally Appropriate Visual Scale

The use of visual imagery in the scoring of the pediatric experience of pain has been well documented and clinically implemented [24,38,67,74,75,80]. In 2002, Magaret et al. [38] utilized a modified Wong–Baker FACES Pain Rating Scale to survey pediatric patients about their satisfaction with their pain management in the emergency department. Despite the widespread use of visual faces scales in the assessment of pediatric pain experience, there is no validated faces scale that has been constructed to specifically address pediatric satisfaction. Faces scales provide one method for gathering subjective information from pediatric patients and have been deemed cognitively appropriate in children as young as 4 years old [67,81]. They are also often preferred over other measures, such as numerical or visual analog scales [8]. We posit that an appropriate measure of satisfaction with pain management for younger children ought to include a faces scale to provide a visual index. However, this is an area of our research that is sorely lacking with conflicting evidence in the pain measurement literature.

While developing a visual scale, key factors to consider include total number of faces, anchor points, and the language used to anchor the scale. Most faces pain scales have either been constructed or revised such that their scoring will be copacetic with a classic 0–5 Likert-type scale or the 0–10 numeric pain rating scale [24]. This allows for a six-face scale with a high level of complexity or a three-face scale with greater simplicity. Determining which of these types of scales—complex or simple—is best suited for a satisfaction measure for children requires further research. Possible factors to consider would be how the number of faces in the scale alters the patients’ satisfaction report and whether a three- or six-face scale would be more cognitively accessible for children.

The anchor faces for pain scales have come under scrutiny in recent studies, with the suggestion that a smiling “no pain” face has the propensity to increase pain scores [23] and a face with tears demonstrating “most pain” can decrease pain scores because children may not want to admit to crying or may believe that crying is a requisite of experiencing the most pain [81]. While these comments do not directly translate to satisfaction research, they certainly draw attention to the importance of choosing anchor points wisely, with the intention of avoiding any pitfalls of misperception. Additionally, young children tend to be better at distinguishing external features of an object than the internal features. Thus, varying the size and/or shape of the faces will perhaps provide a more developmentally appropriate measure [57]. It is important during the construction of a faces scale to ensure that the chosen face images are at evenly spaced intervals from one another [38,67,74]. This will be critical for the data to be translatable into numerical scales for analysis and comparison.

When constructing a visual scale of satisfaction, it will be important to ensure that it is psychometrically sound, carefully constructed, and appropriately validated. The best guide for satisfaction scale development is to draw from the pediatric pain scale literature. Champion et al. [82] and Hicks et al. [67] developed visual scales for measuring pain (Faces Pain Scale-Revised), and more recently Baxter et al. [83] adapted their methods to develop and validate a pediatric nausea scale (BARF). The methods for developing appropriate pediatric scales include: assembling a dedicated team of experts on the subject in question (pain, nausea, satisfaction, etc.) to discuss the details of what the proposed scale will measure and what dimensions it ought to contain, creating a wide selection of possible faces that include the experts’ suggestions, and testing the tool with a sample pediatric population against a known and validated measure. Hicks et al. [67] and Champion et al. [82] used an adult population to choose four faces internal to the anchors (for a total of six faces) from a wide selection showing a steadily increasing severity of pain. Baxter et al. [83] used a similar method—they then went on to convert the six-face scale selected by their sample population into 140 smoothly morphing faces that represented a scale of no nausea to emesis (maximum nausea). They then had a group of 15 pediatric nurses scroll through and select faces based on the prompting of “20%, 40%, 60%, and 80%” nausea. Using these methods, they could construct a six-face scale that was built on the input of experts, pediatric patients, and pediatric nurses with many years of experience. These studies provide a strong framework with which to develop a sound, validated faces scale to measure pediatric satisfaction.

Given the complexity of the measure of satisfaction, it may be quite difficult to depict an image of satisfaction in a simple two-dimensional image. Indeed, satisfaction has an emotional value ascribed. In fact, the Wong–Baker measure has been criticized for potentially depicting emotion-based pain instead of physical pain as opposed to the FPS-R which shows pain with less emotional valence. During the development of a developmentally appropriate visual analog measure of satisfaction, we should aspire to incorporate all elements of care that are associated with satisfaction.

#### 3.3.2. Considerations for a Developmentally Appropriate Scale

For older youth and adolescents, the satisfaction measures used for evaluation by adults may be appropriate for use. Indeed, the numeric rating scale used to measure acute pain experience by adults is validated for use in children as young as 8 years of age [24]. It has been outlined that satisfaction is multidimensional and complex and it is not clear whether these measures will be appropriate or adequate. Investigations are needed, particularly in the acute care setting, to determine if older youth and adolescents are able to use the measure. The aspects of care that drive their satisfaction will also be interesting to explore given the prior literature showing differences in the reporting of satisfaction between pediatric patients and their parents [12,13,38,50].

### 3.4. Choosing the Appropriate Anchor Language

A small number of studies have been performed to elucidate the language children use when describing the concept of pain. The most frequent words used by children from age 1 to 9 to describe pain were “hurt”, “ouch”, and “ow”, with the latter appearing as early as 17 months of age [15]. Most children understand the meaning of pain by 6 years of age [15]; however, some children as old as 11 years of age have difficulty with the concept [84,85]. This demonstrates the importance of utilizing developmentally appropriate language when formulating a satisfaction measurement tool such that all children may be granted a voice in their healthcare assessment.

Similar research has been performed to discuss the language of satisfaction in young children aged 3 to 18. The words “good”, “happy”, and “better” were used to express satisfaction with pain management in a pediatric emergency department [86,87]. “Good” and “happy” were used by children at all developmental levels. The words “sad”, “bad”, and “mad” were most commonly used to describe dissatisfaction; however, there was much greater variability with the negative responses than with the positive. As children progressed cognitively, their answers became more diverse and complex, but rarely reached a point where they spontaneously used the term “satisfaction” while assessing their pain management. In fact, only children older than 13 years of age can reliably understand the meaning of satisfaction [87]. As such, in any construction of a satisfaction measure, it is vitally important to use language anchors that will close the gap and allow for utilization of the instrument with younger children.

### 3.5. Integration across Health Networks

In a diverse healthcare system, where needs and outcome measures differ both between departments and between hospitals, developing and adopting a measure that is valid and reliable across health networks should be prioritized. To this end, simplicity will allow for an effective and replicable measure that can complement department- and population-specific satisfaction quality assurance processes with little added burden. Ideally, the measure would be instructive in circumstances of acute and chronic pain management, and in both inpatient and outpatient settings. Using the same tool across settings, age groups, and institutions will allow for meaningful comparisons, quality monitoring, and ultimately improvements in the care provided.

## 4. Conclusions

This paper has synthesized what is known about both satisfaction and pain management as it pertains to pediatric patients. Due to the relative dearth of research in this critical area, it is not a traditional literature review paper, but rather a framework that lays the foundation for future research into the development of a satisfaction tool for pediatric pain management. Several fundamental suggestions based on the distinct but connected constructs of satisfaction and pain management were developed with the goal of engaging more researchers and healthcare professionals in the development of a cohesive, efficient, and reliable instrument for encouraging assessment via self-reporting from children. This discussion should solidify the importance of this critical area of research, expanding on the PedIMMPACT call-to-action from 2008, and help guide the creation of a developmentally appropriate tool to measure children’s satisfaction with acute pain treatment.
